# Predictors of retirement satisfaction in the older adults of Urmia: a cross-sectional study

**DOI:** 10.1186/s12877-022-03259-1

**Published:** 2022-07-05

**Authors:** Mojtaba Honarvar, Javad Rasouli, Jamileh Amirzadeh-Iranagh

**Affiliations:** 1 Social Determinants of Health Research Center, Clinical Research Institute, Urmia University of Medical Sciences, Urmia, Iran; 2 Department of Public Health, School of Public Health, Urmia University of Medical Sciences, Urmia, Iran

**Keywords:** Retirement satisfaction, Quality of life, Life satisfaction

## Abstract

**Background:**

Retirement is a challenge that, as a process, influences the individual’s role, status, life patterns, expectations, and available resources. Therefore, the present study aims at determining the predictors of retirement satisfaction among men in the city of Urmia.

**Methods:**

In this descriptive-analytical study, 140 retired men living in Urmia were selected by multi-stage sampling method. The instruments used are the Retirement Satisfaction Scale, life satisfaction, and quality of life questionnaires. Data were analyzed in SPSS v.21 using descriptive statistics, one-way ANOVA, and regression at a significance level of *p* ≤ 0.05.

**Results:**

Based on the results of this study, the mean score of retirement satisfaction was 115.37 + 10.13 and there was no significant difference (*p* = .068) in retirement satisfaction of the retired men based on level of education. Also, the retrospective multiple linear regression model indicated that 44.4% of the variance of retirement satisfaction score is predicted by two subscales of life satisfaction and quality of life.

**Conclusion:**

According to the results, it seems that life satisfaction and quality of life are inseparable, effective factors in retirement satisfaction, so, to promote retirement satisfaction in all of its scales and subscales, it is recommended to improve these two factors.

## Background

Today, the world’s population is aging and the elderly population is projected reach two billion in the next 50 years [1]. Iran is one of the ten countries with an aging population and will face an aging crisis in the next 30 years [2]. In the meantime, the phenomenon that grows with old age is retirement. Retirement and old age are often interrelated and occur in a temporal symmetry with one another [3]. Retirement is associated with reduced psychological commitment and behavioral withdrawal from work. This definition is consistent with the evolutionary theory of life, which states that retirement is a stage that not only corresponds to a decrease in activity and physical productivity, but also includes a reduction in responsibility towards others in daily life. Thus, the aging process often coincides with the transition from working to retirement [4]. A review of global demographic forecasts shows that by 2050, more than 20% of the U.S. population will be at or above 65, up from about 13% in 2010. This will lead to a significant increase in the number of people transitioning from working to retirement in the next four decades [5]. In Indonesia the number of retirees will reach 62 million [6]. Brazil, Mexico, Argentina, Turkey, Russia, Britain, France, China and South Korea are also expected to experience a similar situation in 2050. Iran is no exception, and in the next thirty years, a quarter of the whole population will be made up of retirees [7]. As a social phenomenon, retirement is an important factor that inevitably occurs in the working life of humans, which may also occur in premature periods for various reasons. This phenomenon has social and economic dimensions and consequences [8].

Retirement is a transformation from one role to another that creates deep interruptions in the individual’s life. So, how a person reacts to retirement as well as his ability to cope satisfactorily with this fundamental issue has a great impact on the health of the older adult [9]. Retirement can change patterns of life and affect the identity of retirees [10].

Retirement is also a time of losing relationships, which not only means losing the job, but it also losing contact with those in the workplace [11]. These changes create a sense of loneliness in retirement and affect retirement satisfaction [12]. Obviously, retirement satisfaction is something related to mental health during the old age. Retirement satisfaction is the level of satisfaction and happiness of a retiree at a certain stage of life due to physical and mental conditions. Another factor that can affect retirement satisfaction is the economic status. Retirement actually involves a reduction in income [11]. Some people may be re-employed during the old age due to economic, social and psychological problems caused by lack of attention to their demands, living expenses and lack of social support, which in turn causes the older adult to have less time to rest, communication with others, family and leisure time, which affects a person’s mental health [13]. On the other hand, it has been shown that people who are forced to retire are 30.2% less likely to have the highest level of satisfaction, but in those who have lower obligations to retire, which value is decreased to 20.6% [14]. One of the studies in this field, which aimed at the assessment of the retirement satisfaction in the retired employees of Saderat Bank in Kohkiluyeh and Boyer-Ahmad, indicates that the retirement satisfaction of retirees was at a low level [15]. In another study, Kianfar et al. reported that there is a positive correlation between good financial situations and satisfaction with retirement, but a significant negative relationship between gender and retirement satisfaction. In other words, the older men are less satisfied with their retirement than older women [16]. The main reason is that in Iran, retired women, in addition to their monthly income also benefit from their spouse’s salary or Survivors ‘pension at the same time, which can make comparing the quality of life with retirees’ life satisfaction difficult. Due to this, it was decided to examine retired men who provide the family income alone in this study. Some studies have identified gender index, life satisfaction, quality of life, social factors, family and marital relationships, and financial resources as influencing retirement satisfaction. Life satisfaction and quality of life (QOL) may influence retirement satisfaction, among other factors. QOL is a multidimensional concept relating to an individual’s perception of their life situation according to their culture and values. It comprises domains such as living standards, well-being, economic independence, and housing requirements [17]. Additionally, life satisfaction is a particular concept that is best described as a cognitively orientated, subjective view of one’s current life situation compared to one’s expectations [18]. Three hypotheses were designed for the present study based on the above.

1. Individual characteristics and social factors predict retirement satisfaction.
2. Quality of life predicts retirement satisfaction.
3. Life satisfaction predicts retirement satisfaction.

## Methods

The present study was a descriptive-analytical study. The study population consisted of male retirees living in Urmia and the sample size consisted of 140 governmental and non-governmental retired employees who were selected by cluster sampling. Lack of history of chronic diseases with a disability, age range over 60, retired male with an unemployed spouse, consent, and willingness to participate in the study were the inclusion criteria, and the Alzheimer’s disease and history of memory-related diseases were the exclusion criteria of the study. All subjects completed the 51- item Retirement Satisfaction Scale [16], a Life Satisfaction Questionnaire with a 20 - point Likert response format [19], and a Quality of Life (Lipad) Questionnaire with a 30 - item. One score was given for each item of the questionnaire. Then overall mean scores were calculated for questions. Also, the demographic characteristics questions included personal characteristics such as age, retirement age, retirement period, marital status (single, married), number of children, employment status of the spouse, and willingness to retire. Using RSS’s approved questionnaire in this study, Cronbach’s alpha and halving methods were used to determine the reliability of the questionnaire, and values of 0.89 and 0.88 respectively were obtained, showing that the questionnaire was reliable. To determine the validity of the questionnaire, its total score was correlated with the score of the benchmark question and it was found that there is a significant positive relationship between them (*P* = 0.0001 and 0.76).

With the results of the study of Lewis et al. [20] and based on the following formula, the sample size was calculated to be 140 subjects.

$${\left(\frac{Za+ Z\beta}{C}\right)}^2=n=140$$

$$C=.5\times Ln\left[\left(1+r\right)/\left(1-r\right)\right]$$

1-β: Power of the test = 0. 05

 r: Sample correlation = 0. 44

 Study design effect = 1. 7

 Attrition rate=. 15

 α: Significance level (two sided test) = 0. 05

In order to conduct the study, after receiving a referral letter from the Vice Chancellor for Research in the Urmia University of Medical Sciences and Health Services and making the necessary arrangements with the Urmia Health Center, and after obtaining permission, sampling was conducted among comprehensive health centers using a multi-stage sampling method. Thus, at first, the city of Urmia was divided into two districts of north and south. There were 18 comprehensive health centers in the northern district and 8 in the southern district. Among these centers, 9 centers were randomly selected from the north and 4 from the south. Then, in each cluster, a list of eligible individuals was extracted and individuals were selected using a random number table. They were invited by phone calls to complete the questionnaire. The researcher introduced himself to the participants, explained the objectives and methodology of the study, and obtained the subjects’ consent to participate in the study. The questionnaires were read and completed by the two researchers trained in the subject.

After collection, data were entered into SPSS v.21. After confirming the normality of the data, descriptive statistical methods (mean and standard deviation) and analytical tests (Pearson correlation coefficient, one-way analysis of variance) were used in the Kolmogorov-Smirnov test. Multivariate analysis (linear multivariate regression) was used to evaluate the predictive power of independent variables and to match and control the effects of interfering variables. Statistical analyzes were performed at a significance level of 0.05.

### Results

Out of 140 retirees, 32 had an educational degree of below diploma, 50 (35%) had a diploma, 27 (19.3%) had an associate degree and 31 (22.1%) had a master’s degree or higher. The minimum age of retirees was 60 and the maximum 86 with a mean of 65.14 ± 5.404 and the elapsed time since their retirement was a minimum of one year and a maximum of 40 years with an average of 12.7 ± 278 years. Also, all retirees had a minimum of one child and a maximum of 6 children, and the average number of children among retirees was 3. In this study, to evaluate the retirement satisfaction of the subjects, first the mean and standard deviation of retirement satisfaction scores and its various aspects were calculated according to Table 1. The results indicated that the mean score of life satisfaction was 9.26 ± 2.55, the mean score of spouse’s satisfaction of retirement was 10.6 ± 2.62, the mean score of adaptation to retirement was 8.33 ± 1.63, the mean score of retirement health was 7.35 ± 1.89, health of the retired spouse 7.42 ± 1.83, marital interaction 5.31 ± 1.29, job stress 8.3 ± 2.19, leisure time 14.28 ± 3.54, self-efficacy 11.24 ± 2.29, mental state 6.15 ± 1.31, marriage conflict 14.48 ± 3.59, compulsory retirement 9.18 ± 3.99, the total score of retirement satisfaction 115.37 ± 10.13. According to Table 2, there is no significant difference in retirement satisfaction of the older man based on level of education (*p* = .068).

**Table 1:** Descriptive statistics of the aspects of retirement satisfaction of the retired men

Aspects of retirement satisfaction	Min	Max	Mean	SD
Life satisfaction	4	15	9.26	2.55
Spouse’s retirement satisfaction	4	16	10.6	2.62
Adaptation to retirement	3	14	8.33	1.63
Health status during retirement	3	12	7.35	1.89
Health status of the retired spouse	4	12	7.42	1.83
Marital interaction	2	8	5.31	1.29
Job stress	3	13	8.3	2.19
Leisure time	4	20	14.28	3.54
Self-efficacy	5	18	11.24	2.29
Mental and psychological state	2	10	6.15	1.31
Marital engagement	5	20	14.48	3.59
Forced retirement	4	17	9.18	3.99
Total score of retirement satisfaction	86	146	115.37	10.13

**Table 2:** Comparison of retirement satisfaction based on the level of education in the retired men

Test^*^	Mean difference (J-I)	Level of education (J)	Level of education(I)	Dependent variable
	1.69	Diploma		
	1.92	Associate	High school	
	6.45	BA & higher		
*p* = .068	0.22	Associate	Diploma	RSS
	4.75	BA & higher		
	4.53	BA & higher	Associate	

* One Way-ANOVA Test; *RSS* Retirement Satisfaction Score

Shown in the Table 3, retirement satisfaction has a moderate relationship with the variables of spouse’s retirement satisfaction (r = 0.58), life satisfaction of the retired person (r = 0.52), leisure time (r = 0.41), health status of the spouse (r = 0.37), health status of the retiree (r = 0.33), a weak relationship with the variables of marital conflict (r = 0.25), mental health of the retiree (r = 0.13), job stress (r = 0.13) and a negative relationship with retirement adjustment (r = − 0.05) and self-efficacy (r = − 0.01).

**Table 3:** Matrix of correlation coefficients of research variables

	1	2	3	4	5	6	7	8	9	10	11	12
1-LSat	1											
2-SpSat	.47**	1										
3-Rcop	−0.09	−.20*	1									
4-Rhth	.23**	0.1	−0.09	1								
5-Shth	.23**	.22*	−0.08	.44**	1							
6-Ci	.23**	.41**	−.27**	0.13	.21*	1						
7-Js	−0.01	0.08	0.12	−.18*	− 0.08	0.03	1					
8-Leist	0.09	0.12	−.23**	0.04	0.08	.32**	−.2*	1				
9- Selfe	−0.01	−.18*	− 0.02	− 0.04	− 0.08	− 0.11	− 0.15	− 0.11	1			
10- Psy	− 0.01	0.07	0.14	−0.02	−0.04	− 0.03	−0.01	0	−0.07	1		
11-Mc	0.01	−0.07	0.15	0.01	−0.14	−.17*	0.05	−0.14	0.11	0.05	1	
12-Tresat	.52**	.58**	−0.05	.33**	.37**	.47**	0.13	.41**	−0.01	0.13	.25**	1

* = 0. 05, ** = 0. 01, *Lsat* Life satisfaction, *SpSat* spouse satisfaction, *Rcop* Coping with retirement, *Rhth* health of retiree, *Shth* health of spouse, *Ci* Couple interaction, *Js* job-related stress, *Leist* leisure Time, *Selfe* Self efficacy, *Psy* Psychological, *Mc* Marital conflict, *Tresat* total satisfaction with retirement

Retirement satisfaction predictors were determined by multivariate regression analysis (Table 4). For this purpose, the dependent variable of retirement satisfaction and independent variables including age, level of education, marriage duration, number of children, sleep disorder, retirement period, retirement satisfaction, quality of life and life satisfaction were included in the model and the analysis was performed using the backward analysis method. The regression model showed that the two subscales of life satisfaction and quality of life could predict 44.4% of the variance of retirement satisfaction score. Therefore, the second and third hypotheses of the research were accepted, while the first hypothesis was rejected.

**Table 4:** Predictors of retirement satisfaction of the retired men

F	Adjusted R^2^	R	*P*-value	t	Beta	B		Model
			0.001	4.5		42.6	constant	
46.932, df1 = 2, df2 = 113, *p* = 0.001	0.444	0.674	0.001	7.46	0.52	1.71	Life satisfaction	7
			0.001	6.22	0.43	0.61	Quality of life	

## Discussion

As part of the present study, the life satisfaction factor was one of the predictors of retirement satisfaction. Accordingly, the second hypothesis was accepted. Retirement satisfaction had a moderate and positive relationship with the variable of life satisfaction, that is, with the increasing life satisfaction in the retired subjects, the retirement satisfaction also increases, although it was not in line with the results of a similar study where there was a negative relationship between the two [21]. In this regard, Shin believes that retirement satisfaction is inevitably related to life satisfaction because retirement is a reflection of a person’s current reality, and in general, with retirement satisfaction, the individual’s life satisfaction also increases [22]. The results of that study also suggest that retirement satisfaction has a great impact on life satisfaction, and specifically, this relation will be more prominent if retirement is accompanied with early elimination from the labor market, post-retirement planning, loss of social/economic roles, declining income, inadequate social welfare, and persistent financial needs for education and marriage of children. In this regard, Heybroek et al. concluded that retirement is not a uniform experience and that changes in life satisfaction during the retirement period are associated with pre- and post-retirement experiences [23]. Gorry et al. emphasized that retirement satisfaction has an effect on life satisfaction, and this effect is immediate and lasts for a long time [24]. Also, a study in Australia showed that life satisfaction increases gradually in the individuals who cope with retirement and are satisfied with it, and conversely, life satisfaction decreases in those who are not satisfied with retirement [25].

Quality of life was the second predictor of retirement satisfaction; therefore, the second hypothesis was also accepted. Examining the factors quality of life, it can be stated that the results are indirectly in line the results of other similar studies. According to the results of Solinge et al., limited financial resources, sharp decline in post-retirement income and reduced health status are predictors of retirement dissatisfaction. In addition, death of the spouse or divorce during retirement as well as forced retirement reduces retirement satisfaction [26]. Also, a study by Fouquereau et al. conducted in six European countries found that the variables of satisfaction with health status and income, marriage and family, and variables of freedom and re-control are predictors of retirement satisfaction [27]. In the study of Taylor et al., the variable of retirement expectations played a pivotal role in predicting retirement and life satisfaction. The most important contribution of this study was that in determining life and retirement satisfaction, expectations were of more importance in the early stages of retirement and their importance diminished over time [28].

Also, in the present study, the relationships between the dimensions of retirement satisfaction were examined, which showed that this factor had a moderate relationship with the spouse’s retirement satisfaction (r = 0.58), which is consistent with the results of Smith et al. They reported that women are three and a half times more satisfied with retirement than their husbands, especially when retirees find their income sufficient for current needs. When retirees leave their works with a post-retirement plan, spouses report significantly higher retirement satisfaction [29].

In the study of retirement satisfaction, there was a negative and very small relationship between this factor and self-efficacy and retirement adaptation dimensions; so, this relationship can be ignored. However, according to Solinge et al., the older adult who have higher scores in self-efficacy are more likely to be easily adapted to retirement [30]. Previous studies on retirement adjustment mostly focused on the economic dimension and financial planning for retirement. Recent studies, however, took a holistic approach, and also focused on factors such as health, leisure activities, and social support as predictors of retirement adaptation [31].

The impact of health status of the couple dimension during retirement on their satisfaction cannot be underestimated, as the results of the current study confirm this belief. In this regard, Handley et al. stated that Australian retirees scored higher in terms of mental health, relationships and life satisfaction than employees [32]. But in a similar study, according to Olesen et al., psychological distress did not differ between subjects of all ages with respect to retirement status [33]. Also, in the study of Byles et al., dissatisfaction with retirement along with mental disorder is evident in men with physical dysfunction [34]. According to Mein et al., comparison of retirees and employees shows that mental health status deteriorates among those who continue to work, but improves among retirees, and that physical health declines among both employees and retirees [35]. A study by Smith et al. also showed that poor physical health is associated with lower retirement satisfaction and poor spouse health has a negative effect on marital quality [29]. In explaining this study, it can be pointed out that the way a person adapts to retirement is affected by health status. Therefore, when a healthy person has sufficient health status during retirement, he will have a higher retirement satisfaction, because he considers himself capable of doing other activities besides his former job [16].

One of the reasons that the older adult enjoy their retirement period is the marital relations. In the present study, there is a positive relationship between the two factors of marital interactions and conflicts with increased retirement satisfaction. Couples who are both retired experience lower marital stress than working or job-seeking couples [36]. Another similar study by Henry et al., which examined 106 subjects in the age groups of 40 to 50 and 98 older adult in the age group of 60 to 70, found that the older adult had higher marital satisfaction and negative interactions between spouses is smaller in this group and some of their positive characteristics are more prominent, specifically that there is less potential for conflict and quarrel, but a higher potential for happiness and enjoyment in this age group [37].

In the present study, the variable of adaptation to retirement had a negative relationship with retirement satisfaction (r = 0.05). These factors can affect adaptation to retirement. According to Schellenberg, & Turcotte, individuals in higher-stress managerial, professional, or technical jobs are much more likely to adapt to their retirement than those in jobs with lower stress. Although the impact of stress on health may be different for men and women complementary models have shown that the correlation between job stress and the likelihood of adaptation to retirement is very similar for both genders [38].

Another factor studied leisure time during retirement, which had a positive and moderate relationship with retirement satisfaction. In the study of Hetherington et al., a significant correlation was found between retirement satisfaction and leisure time adequacy [39]. Proper pastime is essential for the well-being of the older adult. Activity, exercise and entertainment increase the life satisfaction of the older adult, strengthen their self-confidence and save them from inertia, fruitlessness and feelings of being ignored. Knowing how the older adult spend their leisure time reveals their real needs and helps planners to be consistent with their wishes and needs [40].

## Conclusion

Due to the increasing number of older adult in developing countries, especially Iran, there is a higher need for organized planning by officials and activists in the field of aging. Based on findings of the present study, it seems that, in order to improve retirement satisfaction, policymakers should pay more attention to the quality of life and life satisfaction of the older adult.

## Limitations

This study was carried out among male retirees. Therefore, due to cultural, linguistic, and ethnic differences, caution should be exercised in generalizing these findings to older women and subjects living in other parts of the country. It would be beneficial to conduct further research to extract other significant factors that can be used to predict retirement satisfaction in older adults using both quantitative and qualitative research methods. In addition, it was not possible to confirm the hypotheses with a more robust data analysis.

## Data Availability

All datasets generated and collection tools or analyzed during this study are available from the corresponding author on reasonable request.
